# Artificial Intelligence (AI)-Driven Adaptive Motion Management in Helical and Robotic Radiotherapy: Innovations, Challenges, and Future Directions

**DOI:** 10.7759/cureus.81702

**Published:** 2025-04-04

**Authors:** Dhiren K Panda, Saurjya R Das, Shishir Kumar

**Affiliations:** 1 Anatomy, Institute of Medical Sciences (IMS) &amp; SUM Hospital, Siksha 'O' Anusandhan University, Bhubaneswar, IND

**Keywords:** adaptive radiotherapy, ai-driven motion tracking, cyberknife motion management, deformable image registration, real-time tumor tracking

## Abstract

Artificial intelligence (AI) has revolutionized motion-adaptive radiotherapy (ART) by enhancing tumor-tracking accuracy and optimizing radiation dosage delivery. Traditional motion management techniques, such as respiratory gating and internal target volume (ITV) expansion, often result in increased treatment margins and unintended radiation exposure. AI-powered real-time motion tracking, deformable image registration (DIR), and ART offer superior tumor localization, automated dose modulation, and real-time imaging integration.

This study examined AI-based motion guidance technologies in helical tomotherapy (HT) and CyberKnife (Accuray, Madison, WI) robotic radiosurgery, highlighting technical innovations, engineering challenges, and clinical applications. HT employs megavoltage computed tomography (MVCT) for intra-fractional motion monitoring, whereas CyberKnife utilizes x-ray-based beam correction via a robotic arm to achieve submillimeter precision. Despite advancements, challenges such as AI processing latency, tumor motion variability, and multimodal imaging integration persist. Future research should focus on improving the AI response times, enhancing motion prediction algorithms, and developing fully automated AI-based radiation delivery systems.

## Introduction

Radiotherapy (RT) is effective only when radiation doses targeting the tumors are delivered accurately and surrounding normal tissues are spared. The mobility of tumors as a result of breathing, heart activity, and gastrointestinal peristalsis are important hindrances that cause dosimetric uncertainty and geometric differences between the preset and actual radiation doses [1,2]. Motion leads to several millimeter shifts in the position of highly mobile tumors, such as those located within the lungs, liver, pancreas, and prostate, thus increasing the prevalence of both inadequate tumor coverage and accidental delivery of radiation to surrounding organs [3].

A wide range of motion control techniques has been devised to address these issues. Although they are useful for compensating for tumor motion, conventional methods, such as internal target volume (ITV) expansion, respiratory gating, and deep inspiration breath-hold (DIBH), tend to induce unintentional radiation exposure [4,5] while increasing treatment margins and prolonging treatment duration. New dynamic motion compensation using recent advances in artificial intelligence (AI), real-time imaging, and adaptive RT (ART) has increased the accuracy of tumor tracking and radiation dose adaptability [6,7].

HT and CyberKnife (Accuray, Madison, WI) are among the most widely used ART modalities with real-time motion monitoring and adaptive dosage modulation. CyberKnife compensation for submillimeter motion is enabled by X-ray image-guided tracking and robotic-beam correction [8,9]. HT uses megavoltage computed tomography (MVCT) imaging for treatment delivery [8,9]. Additional enhancement of these systems has been achieved through the incorporation of AI-based deformable image registration (DIR) and deep learning-based motion prediction, permitting automated adjustment of the beams to accommodate tumor motion [10,11]. However, clinical and engineering challenges remain unaddressed.

Latency in AI-based motion compensation

AI models necessitate response times of less than 50 milliseconds for real-time adaptation; however, computational delays can compromise precision [12,13].

Inter- and intra-fraction motion variability

Tumor motion patterns vary between treatment sessions and even within the same fraction, complicating the implementation of generalized tracking models [14].

Integration of multi-modal imaging and AI

The combination of four-dimensional (4D) MRI, CBCT, and MVCT for real-time motion tracking requires high-speed processing, seamless software interoperability, and robust data fusion techniques [15,16]. This study explored engineering solutions and clinical applications of AI-embedded motion control in HT and CyberKnife systems. It offers insights into the future of AI-enhanced automated RT, describing advancements in motion-tracking algorithms, real-time image incorporation, and adaptive radiation dose management [17,18].

This study details an adaptive AI motion management technique for helical tomotherapy (HT) and CyberKnife systems. Our method differs from standard methods, such as ITV and 4D-CT, which are based on set margins, in that it allows for real-time motion tracking together with dynamic adaptation. The incorporation of AI into automated motion assessment will enhance treatment accuracy, reduce unnecessary radiation dose, and improve clinical workflow efficiency. This study contributes to the existing literature by overcoming some shortcomings of conventional motion management techniques and relative RT.

## Technical report

Motion prediction and tracking in ART

Adjustments for organ movement during treatment are a persistent problem in RT, especially for lesions located in the chest and abdomen. A solution may be provided through real-time AI adaptation, which analyzes and corrects movements before and throughout the treatment. The next sections will explain how these developments have been achieved, enabling less reliance on traditional ITV margins and periodic imaging [1,2]. To overcome these constraints, motion-ART uses real-time tumor-tracking technologies that continually monitor and compensate for intra-fractional and inter-fractional motion. Different techniques are used in HT and CyberKnife robotic radiosurgery to provide motion correction, thereby guaranteeing accurate radiation treatment of moving tumors [3].

HT and motion-tracking mechanisms

The HT was equipped with a fan-beam MVCT imaging device that continuously scanned the patient during treatment. This method provides real-time anatomical data that can accommodate tumor motion through intra-fractional changes. Combined with adaptive dose modification methods, the radiation beam is still directed to the tumor within organ motion and positioning variations [4,5].

HT utilizes an MVCT-based tracking system integrated with multileaf collimators (MLCs) to enhance the radiation accuracy. This system enables pretreatment and intra-fraction imaging for tumor positioning verification, facilitates dose recalculations during treatment to compensate for motion-induced displacement, and dynamically adjusts the radiation beam in real-time as the tumor moves [6].

CyberKnife and real-time image-guided motion compensation

In contrast to HT, CyberKnife® robotic radiosurgery employs non-isocentric robotic arm technology that dynamically adapts the radiation beam angles based on tumor motility. CyberKnife system: X-ray-based tracking cameras incorporated into the CyberKnife system continuously tracked the position of the tumor being treated. The CyberKnife device offers the advantage of continuous beam modulation without breath-holding or forced breathing [7] and is not possible in conventional dating methods.

The motion-adaptive aspects of CyberKnife include x-ray tracking with submillimeter accuracy for motion localization, dynamic robotic beam delivery, which is an adjustment that occurs according to the patient's movement, and fiducial-free tracking of specific tumor sites, thereby alleviating discomfort to patients [8]. HT and CyberKnife are important advances in motion-ART, although they utilize different tumor tracking and dose-adaptation strategies.

Real-time imaging for motion management

Modern motion-ART platforms rely on advanced imaging techniques to improve tumor tracking accuracy. Several imaging modalities have been integrated into RT workflows to enable continuous tumor localization and adaptive radiation delivery. The imaging techniques discussed above are summarized in Table 1.

**Table 1:** Imaging modalities used in motion-adaptive radiotherapy 4D cone-beam CT (CBCT): Enables volumetric imaging of tumors in motion, allowing clinicians to track respiratory-induced displacement during treatment. Megavoltage CT (MVCT): Used in helical tomotherapy to provide high-contrast imaging for real-time anatomical guidance and treatment verification. MRI-linac: Integrates magnetic resonance imaging (MRI) with radiotherapy to continuously track soft-tissue changes without requiring fiducial markers. X-ray tracking (CyberKnife): Uses orthogonal x-ray imaging to continuously monitor tumor position, enabling fiducial-free motion tracking with sub-millimeter precision.

Imaging modality	Platform	Application in motion management	Advantages
4D cone-beam CT (CBCT)	CyberKnife, LINAC	Captures tumor motion during treatment	High spatial resolution
Megavoltage CT (MVCT)	Helical tomotherapy	Provides real-time anatomical guidance	Enables intra-fraction motion correction
MRI-linac	MRI-Linac	Tracks soft-tissue motion continuously	No need for fiducial markers
X-ray tracking	CyberKnife	Fiducial-free real-time tumor tracking	Sub-millimeter accuracy

Each of these imaging techniques contributes to improved real-time tumor visualization and radiation dose adaptation, reducing uncertainties caused by motion.

Beam adaptation and automated dose modulation

In addition to motion tracking and imaging, beam adaptation technologies are integral to motion-ART.

MLC Tracking for Dynamic Beam Shaping

MLCs allow HT and CyberKnife to shape the radiation beam dynamically. These MLCs change in real-time, improving dosage conformance to the tumor site [14]. Particularly for cancers with complicated motion patterns, MLC tracking improves dose distribution precision, dynamically adjusts the beam aperture to match tumor movement, and reduces motion-induced dose errors, thereby enhancing treatment accuracy [15].

Gating vs. Continuous Motion-Tracking Techniques

Although respiratory gating has been widely used to match radiation with breathing cycles, continuous motion-tracking methods now offer improved accuracy without requiring treatment interruptions [16]. HT uses motion-adaptive MVCT scanning to change the treatment strategies between fractions. Using real-time corrections, CyberKnife technology constantly changes the beam positions, eliminating the need for gating.

Automated Dose Recalculation and Adaptive Beam Modulation

HT and CyberKnife used computerized dose recalculation to change the radiation distribution in real-time [17]. These systems ensure that the dosage remains within the intended tolerances by analyzing tumor movements and avoiding underdosing or excessive radiation exposure.

Final technical report summary

The different helical and cyber-knife therapies are specialized for surgery and utilize different motion tracking strategies to improve the accuracy of treatment. The application of real-time imaging methods further enhances tumor tracking accuracy, enabling more precise dose adjustments. Frequently, MLCs adjust the beam shape to move the tumor optimally dose distribution. Most importantly, robust automatic dose recalculation helps to maintain the precision of radiation delivery control even when continuously tracking moving tumors, which is essential for reliable radiation delivery. Further development is required for motion prediction algorithm optimization and the addition of AI for automatic motor control to improve the treatment efficacy and patient results.

The application of AI in motion management significantly improved ART. Target motion tracking enables real-time dynamic dose adjustments. Consequently, treatment margins, conformity, and precision were improved. Future work will examine AI algorithms with predictive accuracy for clinical validation studies to quantify patient outcomes incorporated into patient care outcome studies.

## Discussion

Real-time adaptive motion management in RT is subject to several computational and engineering challenges. AI-driven motion compensation latency is among the most significant limiting factors because real-time tracking systems need to predict tumor movements with response times below 50 ms to ensure correct dosage distribution. However, most AI-based methods for motion tracking have non-negligible computational latency, mainly because of the lengthy input image capturing, processing, and dynamic beamforming times [1,2]. In addition, the time performance of AI models on real-time datasets is constrained by hardware limitations, algorithmic inference time, and integration with established treatment workflows [3].

This heterogeneity in tumor mobility poses a major challenge because it can vary widely between patients and even within the same treatment session. Tumor mobility is modulated by structural deformations, irregularities in the respiratory cycle, and interpatient variability. This presents a challenge for the development of generalized AI-guided tracking models because motion compensation algorithms are forced to adapt to intra-fractional and inter-fractional alterations [4,5]. Personalized AI-driven motion management for specific patients is a novel concept, and basic research needs to be conducted as more comprehensive multi-institutional datasets are required for model training [6].

The integration of multimodal imaging and AI-based tracking systems is an ongoing field of investigation and development. By performing tracking of 4D MRI, cone-beam CT (CBCT), and Megavoltage CT (MVCT) techniques provide additional real-time anatomical monitoring is provided that allows for precise variation of radiation dosages [7]. Nonetheless, the integration of these imaging modalities with AI-based motion-correction approaches relies on abundant computational resources and impeccable software interoperability. The integration of high-speed imaging data with AI-generated motion predictions in real-time is complex because of the need for immediate processing to advise therapy adjustments. Parallel computing, high-speed GPUs, and cloud-based AI models are notable advances that have the potential to overcome this challenge, ensuring faster and more efficient motion-ART workflows [9].

Recently, AI-based methods for motion prediction have significantly improved the precision of radiation tracking. Recurrent neural networks (RNNs) and long short-term memory (LSTM) models are utilized to analyze historical tumor motion patterns, leading to preemptively adjusted beam delivery and future trajectory prediction [10,11]. To account for organ deformation, DIR algorithms are used to dynamically warp the organ, ensuring that the dosage is distributed correctly for anatomical variations [12]. Proposals for real-time tumor localization have also included applications for image segmentation in AI-augmented CNNs to reduce reliance on fiducial markers and obtain more accurate tracking in highly motile tumors [13]. These techniques allow targeting errors to be minimized and tumor coverage to be reliable, especially for sites in the body prone to motion, such as the lungs, liver, pancreas, and prostate [14].

Real-time RT has enhanced treatment accuracy largely owing to the development of automated beam-shaping technologies in combination with motion prediction. The development of MLC tracking technology allows beam shape and intensity to be modulated in real time to minimize the geometric uncertainties produced by tumor displacement [15]. Furthermore, continuous tumor monitoring systems and respiratory gating have improved the dose distribution management by minimizing motion-induced errors with minimal imaging exposure [16]. These technological advances have ensured optimal dose delivery, even for complex, non-rigid tumor motion patterns.

AI-based RC enhancements for RT are anticipated to continue in progression processes with an emphasis on model generalizability, reduced computer latency, and adjusted augmentation of the imaging with regard to AI in the future. The principal objective is to develop AI models that can understand imaging data in real time, achieving response times below 30 ms, which will ensure nearly instant motion compensation [18]. In addition, such AI models must undergo training using extensive multicenter databases to enhance their adaptability to diverse patient anatomies and therapeutic strategies [19]. Moreover, fully autonomous AI-trained RT systems are in development, with real-time adjustments generated by algorithms without human involvement, increasing PAR efficiency and accuracy [20].

Challenges and future directions

Despite the significant progress in motion-ART, several challenges remain. Real-time motion processing can introduce slight delays in beam adjustments, necessitating further optimization to improve the accuracy and precision of the treatment delivery [18]. Tumor motion variability, which is influenced by differences in breathing patterns, organ deformation, and patient positioning, makes it challenging to develop a standardized tracking solution applicable to all patients [19]. In addition, processing large volumes of imaging data for 4D motion tracking imposes substantial computational demands, requiring high-speed computing capabilities to ensure real-time adaptation and accurate radiation delivery [20]. Ensuring safety and quality assurance is another critical challenge because fully automated RT workflows must adhere to strict safety standards to maintain reliability in treatment execution [21].

Future advancements should focus on enhancing motion-tracking models to improve accuracy and reduce latency, refining adaptive beam modulation techniques for better tumor dose conformity, and developing fully automated AI-integrated RT workflows to minimize manual intervention. These innovations will further improve treatment precision and clinical outcomes, particularly for motion-sensitive tumors, such as lung, liver, pancreatic, and prostate cancers [22]. This research examines the technical feasibility of implementing AI motion management, whereas future work will include clinical case studies spanning the thoracic, pelvic, and abdominal regions to assess the practical benefits of this technique.

## Conclusions

Motion ART may be substantially improved through the use of AI - facilitated motion tracking and up-to-date imaging, combined with automated adaptation of the beam. AI-assisted motion prediction, DIR, and 4D imaging increase the accuracy of tracking tumors and the precision of delivering radiation doses while minimizing the irradiated volume of healthy organs. Despite these developments, there are still hurdles, such as latency between AI-driven correction and actual delivery, inter-fraction variability of tumor motion, and integration of multimodal imaging. Further studies focusing on accelerated AI processing, adaptive imaging, and complete automated RT workflows are needed to increase treatment accuracy. AI-based motion management will pave the way for the future of real-time ART with higher accuracy, efficiency, and personalization of radiation for motion-sensitive tumors, such as those of the lungs, liver, pancreas, and prostate.
